# The Symmetry of Children’s Knees Is Linked to Their Adult Sprinting Speed and Their Willingness to Sprint in a Long-Term Jamaican Study

**DOI:** 10.1371/journal.pone.0072244

**Published:** 2013-08-19

**Authors:** Robert Trivers, Brian G. Palestis, John T. Manning

**Affiliations:** 1 Department of Ecology, Evolution and Natural Resources, Rutgers University, New Brunswick, United States of America; 2 Department of Biological Sciences, Wagner College, Staten Island, New York, United States of America; 3 Applied Sports Technology Exercise and Medicine Research Centre, Swansea University, Swansea, United Kingdom; University of Goettingen, Germany

## Abstract

Jamaican athletes are prominent in sprint running but the reasons for their success are not clear. Here we consider the possibility that symmetry, particularly symmetry of the legs, in Jamaican children is linked to high sprinting speed in adults. Our study population was a cohort of 288 rural children, mean age 8.2 (±1 SD = 1.7) years in 1996. Symmetry was measured in 1996 and 2006 from the fluctuating asymmetry (FA) of three lower-body traits and we constructed a lower body composite FA trait (Comp lb-FA). In 2010 we measured sprinting speed (for 90 m and 180 m races) in participants recruited from our original cohort. There were 163 untrained adults in our sample. We found: (i) high Comp lb and knee symmetry in 1996 and 2006 were linked to fast sprinting times in our 2010 runners and (ii) our sample of sprinters appears to have self-selected for greater symmetry. We conclude that high knee symmetry in childhood is linked to an ability to sprint fast in adult Jamaicans as well as a readiness to sprint.

## Introduction

Caribbean sprinters, particularly Jamaican sprinters, have long been prominent in elite sprint running [1]. However, the reasons for their disproportionate success relative to the population size from which they are drawn are unclear. For example, ethnic comparisons of ACTN3 and ACE genotypes [2], mitochondrial haplotypes [3] vertical jump and stride number/height ratio [4], and proportions of fast-twitch muscle fibres [5], [6] have been made with mixed success.

Here we take a different approach and focus on long-term symmetry, in particular symmetry of the legs, and its relationship to adult sprinting speed in rural Jamaicans. Our measure of symmetry is fluctuating asymmetry or FA. FA consists of small departures from perfect bilateral symmetry in such paired traits as finger length, knee circumference and foot length [7]. FA has been shown to be a negative correlate of performance rowing, a sport that requires lower and upper body performance [8], and more specifically in sports that emphasise lower body performance, such as sprinting speed in domestic horses (*Equus ferus caballus*) [9] and middle-distance running in humans [10]. With regard to FA in Jamaicans, the Jamaican Symmetry Project (JSP) has focused on long-term correlates of FA in a rural cohort of Afro-Caribbean children [11]. The children were first measured in 1996 when their mean age was 8.2 (±1.7) years and again in 2006. In 1996 nine paired traits were found to show FA (six upper body traits: ear height, finger length [3^rd^, 4^th^ & 5^th^ fingers], wrist and elbow width; three lower body traits: knee and ankle width, foot length). A tenth trait, hand width, showed significant directional asymmetry and was excluded from composite FA indices.

It was found that composite FA of the lower body (Comp lb-FA) was much lower (∼35%) than a composite measure of upper body FA (Comp ub-FA) [11], and comparisons of upper-body FA in this sample with large samples of mostly white children from the United Kingdom show lower FA in the former compared to the latter ([11]–[14], see Table 3 in ref. [11] comparing the 1996 Jamaican data to data from ref. 12 on similarly-aged English children measured using the same protocols). There may also be differences between populations with regard to the magnitude of lower-body FA, but most studies have focused on upper-body traits (e.g., [8], [10], [12]–[14]). Truly comparative data do not exist for lower-body traits, because of differences between studies in the traits that were measured, how they were measured [13], or the age of the subjects [15]. Therefore resolution of this important question will have to await further work.

We tested whether symmetry in children (particularly symmetry of the legs) is related to their running speed when they are young adults. In order to consider this, we recruited adult participants in 2010 from our original 1996 cohort. The original cohort was asked to volunteer for further tests and informed that they would have to complete two sprints. Using the FA’s measured in 1996 and 2006, we then examined the relationships between trait FA’s and sprinting speed recorded in 2010 and also compared the 1996 Comp lb-FA of the participants who did not volunteer to run with those who did. Our prediction was two-fold, i.e. (i) that FA measured in 1996 (and 2006) would be positively correlated with sprinting speed recorded in 2010 and (ii) that those who chose to run in 2010 would have been more symmetrical (particularly in the legs) than those who chose not to run.

## Materials and Methods

The original 1996 cohort consisted of 288 Jamaican children. Of these there were 155 boys and 131 girls (sex recorded by the presence of gender-specific school uniforms, not recorded for 2 subjects), and complete lower body measurements were available for 270 children. Written parental consent was obtained for the original cohort. In 2010 the cohort, now all adults, was contacted by letter (reinforced by word-of-mouth) and informed of the aims of the study. Participants (n = 163) gave written informed consent. The current work and all research in previous years was approved by the Human Subjects Committee of Rutgers University.

In this rural area there was not a conventional running track available. Therefore, we constructed one which was 90 m long. Our two races were 90 m and 180 m long (back and forth on the 90 m track). The second race (180 m) was run about ½ hour after the first race. Subjects were run in pairs (same-sex whenever possible), in order to increase competition during the run. When we had no one with whom to pair with a subject, he or she ran against a dummy runner of the same sex.

Because there was evidence for unequal variances between groups in some cases, we used the unequal variance t-test [16] and df are approximated for all between-group comparisons (analyses performed with IBM SPSS Statistics 21). The dataset has been deposited in the Dryad Repository: http://dx.doi.org/10.5061/dryad.rh8gr.

## Results and Discussion

From the original cohort of 288 children we recruited 163 untrained adults (98 men) in our sample. Means (±1 SD) were as follows: age 22.6 (±1.8) years, sprinting time 14.8 (±3.2) s in the 90 m sprint (n = 163) and 32.5 (±7.3) s in the 180 m sprint (n = 162). Times for the same individuals at the 90 m and 180 m distances were highly correlated (Pearson’s r = 0.87, p<0.0001). Males tended to run faster than females (90 m: males 12.9 [±1.5] s, females 17.6 [±2.9] s, unequal variance t-test [16], t = 11.87, df ∼ 86, p<0.0001; 180 m: males 28.3 [±4.8] s, females 38.8 [±5.8] s, t = 12.13, df ∼ 116, p<0.0001).

The repeatability of signed absolute FA was significant for all traits [11]. With regard to measurements of the original cohort, relative FA (|L−R|/(L+R*0.5) was calculated for each of the traits. Principal Components Analysis revealed five factors that accounted for 61% of the variance in FA’s [11]. An orthogonal transformation showed factor 1 (15% variance) was related to lower body traits (knee r = 0.66, ankle r = 0.68, foot r = 0.64). Factor 2 (13%) was related to 3^rd^ digit (r = 0.73), wrist (r = 0.64), and hand (r = 0.55). The remaining factors were associated with one trait each.

The mean (±SD) relative FA values for the individual lower body traits are as follows: 1996 knee = 0.007 (±0.009), ankle = 0.008 (±0.009), foot = 0.008 (±0.010); 2006 knee = 0.016 (±0.012), ankle = 0.024 (±0.021), foot = 0.020 (±0.017). The composite trait Comp lb-FA was constructed by calculating the mean of all lower body relative FA’s. Comp lb-FA increased from 1996 (mean = 0.008 [±0.006]) to 2006 (0.020 [±0.009]), likely reflecting changes due to rapid growth during adolescence [12]. Comp lb-FA showed no sexual dimorphism in 1996, as males and females had identical means (males n = 147, 0.008 [±0.006], females n = 122, 0.008 [±0.006]), while in 2006 there was a non-significant trend for males (n = 97, 0.019 [±0.009]) to be more symmetrical than females (n = 76, 0.022 [±0.009], t = 1.88, df ∼ 158, p = 0.063).

We removed the influence of BMI from lower body FA by regressing FA (Comp lb-FA, knee FA, ankle FA & foot FA) from 1996 and 2006 on their current BMI and then considered the relationships between residuals (*Res* FA) and sprinting speed. We found that *Res* Comp lb-FA for 1996 and 2006 was positively related to sprinting speed in 2010 (Table 1). Furthermore, a multiple regression analysis with independent variables *Res* knee FA, *Res* ankle FA and *Res* foot FA, showed *Res* knee FA was positively related to sprinting speed in 1996 and 2006 (Table 1). We then calculated mean FA from 1996 and 2006 and regressed this on mean BMI from 1996 and 2006. The residuals from this regression showed significant positive relationships for both *Res* Comp lb-FA and *Res* knee FA to 2010 sprinting times (Table 1). Relationships with knee FA appear to be stronger for the 90 m sprint than for 180 m, but this pattern is not apparent for Comp lb-FA (Table 1). Results are similar if we use raw relative FA values, rather than correcting for BMI (data not shown).

**Table 1:** Relationships (standardized regression coefficients, b) between relative FA independent of BMI and sprinting speed in 2010 for both 90 m and 180 m sprints.

		90 m			180 m		
Year	Trait	b	r^2^	p	b	r^2^	p
**1996**	Res Complb-FA	0.14	0.02	0.09	0.18	0.03	0.03*
	Res FA knee	0.21	0.04	0.01*	0.19	0.03	0.03*
	Res FA ankle	−0.09		0.26	−0.002		0.98
	Res FA foot	0.11		0.18	0.10		0.23
**2006**	Res Complb-FA	0.17	0.03	0.05*	0.17	0.03	0.04*
	Res FA knee	0.24	0.04	0.01*	0.14	0.01	0.10
	Res FA ankle	0.01		0.90	0.07		0.41
	Res FA foot	0.07		0.43	0.09		0.30
**96+06**	Res Complb-FA	0.21	0.04	0.02*	0.21	0.04	0.02*
	Res FA knee	0.30	0.08	0.001*	0.19	0.03	0.03*
	Res FA ankle	−0.04		0.67	0.05		0.61
	Res FA foot	0.12		0.19	0.11		0.21

Sample sizes are as follows: 1996 FA (n = 148 for 90 m, 147 for 180 m); 2006 FA (n = 135 for both sprints); mean of 1996 and 2006 FA (n = 124 for both sprints).

*Note*. Res: Residual; Comp lb-FA: composite lower body fluctuating asymmetry; Statistical significance is indicated by asterisks; r^2^ values are adjusted for the presence of >1 variable when using multiple regression and apply to the overall model rather than any individual trait.

Our participants in the 2010 sprints numbered 163 individuals from our original cohort of 288 children. We considered whether these volunteers showed evidence of self-selection for lower body symmetry by comparing 1996 Comp lb-FA of runners and non-runners in 2010. We found that runners had lower Comp lb-FA (mean = 0.0066) than non-runners (mean = 0.0089, p = 0.006, Table 2). This finding suggested that children with symmetrical lower bodies were more likely to engage in sprinting when adults than children with more asymmetrical lower bodies.

**Table 2:** Mean 1996 composite lower body FA for volunteers (in bold) and non-volunteers for recruitments across years.

Year	Meanlb-FA	N	SD	SE	t	∼df	p
**1996**	**0.0076**	270	0.0062	0.0004	N/A		
**1998**	**0.0073**	139	0.0060	0.0005	0.61	263	0.54
	0.0078	131	0.0065	0.0006			
**2002**	**0.0072**	173	0.0062	0.0005	1.26	197	0.21
	0.0082	97	0.0063	0.0006			
**2004**	**0.0070**	187	0.0059	0.0004	2.14	138	0.034*
	0.0088	83	0.0068	0.0007			
**2005**	**0.0070**	151	0.0063	0.0005	1.69	257	0.092
	0.0083	119	0.0061	0.0006			
**2006**	**0.0072**	168	0.0060	0.0005	1.33	197	0.19
	0.0082	102	0.0066	0.0007			
**2010**	**0.0066**	158	0.0052	0.0004	2.78	187	0.006*
	0.0089	112	0.0073	0.0007			

*Note*. We used the unequal variance t-test and df are approximated [16]. Statistical significance is indicated by asterisks.

In addition to the initial recruitment of our cohort in 1996 and the 2010 sprinting recruitment, there have been five other recruitments (1998, 2002, 2004, 2005, and 2006) which have focused on a variety of target variables. Although mean 1996 lower body FA for participants was lower than the mean for the entire 1996 cohort in each year, this was only significant in 2004 and 2010 (Table 2). In 2004 the target trait was dancing ability.

We have the following findings: Our original cohort of rural Jamaican children was measured in 1996 at a mean age of 8.2 (±1.7) years, and some 14 years later we attempted to recruit the entire cohort in order to measure sprinting speed. We found our new sub-set of participants showed evidence of self-selection. Symmetry of the legs (measured in 1996) was greater in our 2010 sample of volunteers in comparison to the non-volunteers of that year. This effect suggests that Jamaican children with high symmetry in the legs will readily take part in sprints when they are young adults. We think the importance of this finding relates to the enjoyment of sprinting. In addition, although our sample was untrained, this evidence of self-selection may map on to a readiness to train for sprinting events. A similar effect was found in 2004 when participants were asked to volunteer for dancing. We suggest that the traits necessary for successful sprinting may in part map on to traits that are important in dancing. More sedentary target traits were tested in 1998, 2002, 2005, and 2006. In these years volunteers and non-volunteers did not show significant differences in Comp lb-FA.

We found that lower body FA measured in 1996 and 2006 was positively related to sprinting performance in 2010. This was found for Comp lb-FA, and the effect was driven by the association between knee FA and sprinting times. That is, children with symmetric knees in 1996 and 2006 ran faster than children with asymmetric knees when tested for sprinting speed in 2010. Although knees are invariably the key factor, foot size is always a positive co-factor, while ankle width has smaller effects that may go in either direction (Table 1). Knees were also slightly more symmetrical than the other lower body traits.

There are clear links here between childhood symmetry of the lower body and both motivation to sprint and ability to sprint well among rural Jamaicans. The effect is all the more remarkable for the intervening period of 14 years between the first measurements of leg symmetry and self-selection and performance effects related to sprinting. We suggest that high symmetry in the legs–especially knees–may underlie at least some of the advantage that Jamaican sprinters enjoy over their competitors, but comparable measurements of lower body symmetry in other populations are clearly needed. Jamaicans are highly genetically diverse [17], [18] and symmetry should be positively correlated with heterozygosity. However, evidence for a link between heterozygosity and symmetry has been weak or mixed [19]. Furthermore, a general effect of heterozygosity on asymmetry in Jamaicans would not explain a specific effect on knee symmetry. This effect is more likely to have arisen during thousands of years of selection in West Africa and hundreds among their highly heterozygous slave descendents in the New World [18].

If Jamaicans are shown to have more symmetrical legs than those in other populations, we cannot exclude the possibility that they participate in sprinting more often than do others and that this increases the symmetry of their legs. However, the average age of our children was only 8.2 when first measured. This is about the age at which sprinting first becomes common (and then competitive), so we doubt such an effect. In addition, there is no increase in symmetry with age within the 1996 sample (data not shown). It does seem likely though, that peoples of West African origin have been subject to strong natural selection for speed over short distances, and this has influenced a number of traits including developmental stability of the knees and of associated traits in the legs, such as proportion and quality of fast-twitch muscle fibres [1], [5].

It is important to emphasize that degree of knee symmetry is not only a measure of a variable expected to affect speed of running directly, but FA is also a measure of a deeper trait, the developmental stability (under random environmental pressures) of that portion of the body–whether knee symmetry or optimal proportion and function of fast-twitch muscle fibres in nearby leg muscles. Correlations between FA in one variable and other traits are well known in humans, e.g. FA of a-b ridge count and tendency toward schizophrenia [20]. Whether this is true here awaits further study.
